# An accident waiting to happen? Exposing the potential of urogenital schistosomiasis transmission in the Lake Albert region, Uganda

**DOI:** 10.1186/s13071-023-06017-3

**Published:** 2023-11-03

**Authors:** Julius Tumusiime, Grace Kagoro-Rugunda, Casim Umba Tolo, Daisy Namirembe, Ruben Schols, Cyril Hammoud, Christian Albrecht, Tine Huyse

**Affiliations:** 1 Department of Biology, Mbarara University of Science and Technology, Mbarara, Uganda; 2 Institute of Animal Ecology and Systematics, Justus Liebig University Giessen, Giessen, Germany; 3 Department of Biology, Royal Museum for Central Africa, Tervuren, Belgium; 4 Laboratory of Aquatic Biology, KU Leuven, Campus Kortrijk, Kortrijk, Belgium; 5 Department of Biology, Ghent University, Ghent, Belgium

**Keywords:** Lake Albert, Schistosomiasis, Emerging disease, *Bulinus globosus*, *Bulinus nasutus productus*, *Schistosoma haematobium*

## Abstract

**Background:**

Urogenital schistosomiasis caused by the parasitic blood fluke *Schistosoma haematobium* is the most common form of that constitutes a majority of over 240 million schistosomiasis cases. The enigmatic absence of urogenital schistosomiasis in Uganda has, until now, been attributed to the absence of substantial populations of suitable snail intermediate hosts.

**Methods:**

Malacological surveys were carried out in 73 sites southeast of Lake Albert, Uganda in October and November 2020. Collected snails were transported to the laboratory for identification. The snails were identified using partial mitochondrial cytochrome *c* oxidase subunit one and nuclear internal transcribed spacer barcoding. Schistosome infections in snails were also assessed using cercarial shedding and rapid diagnostic PCR techniques.

**Results:**

We found *Bulinus globosus* and *Bulinus nasutus productus*, the main intermediate species in the transmission of *S. haematobium* in mainland East Africa. In this survey, *B. globosus* was more common than *B. nasutus productus*, with the former reported at four sites (total count = 188) and the latter reported at one site (total count = 79). Molecular testing revealed a high prevalence of *Schistosoma bovis* in *B*. *nasutus productus* (16%), but no *S. haematobium* infections were found.

**Conclusions:**

Given the abundance of snail hosts and the risky human water contact behaviours observed, we highlight the potential for urogenital schistosomiasis transmission in the region.

**Graphical Abstract:**

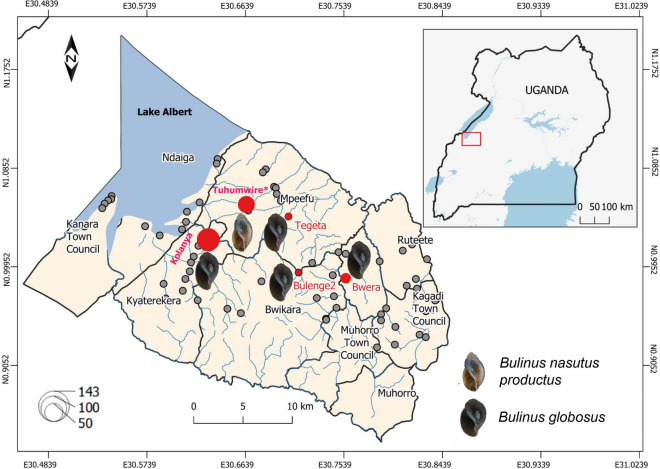

**Supplementary Information:**

The online version contains supplementary material available at 10.1186/s13071-023-06017-3.

## Background

Schistosomiasis is a debilitating neglected tropical disease caused by blood flukes of the genus *Schistosoma.* Over 240 million people are estimated to infected with schistosomiasis [1], with *Schistosoma haematobium*, the causal agent of urogenital schistosomiasis, responsible for 67% of the cases worldwide [2]. Urogenital schistosomiasis infection can have severe health consequences, including anaemia, stunted growth in children, kidney and urinary tract complications, infertility, and an increased risk of human immunodeficiency virus (HIV) infection [3]. Certain species of freshwater snails, such as *Bulinus globosus*, *Bulinus nasutus nasutus*, *Bulinus nasutus productus*, *Bulinus africanus* and *Bulinus truncatus* can act as an intermediate host of *S. haematobium* [4, 5]*,* and their distribution determines where *S. haematobium* infections are likely to occur. Climate change is predicted to drastically alter the geographical distribution of *Bulinus* snails and, consequently, that of their infecting parasites [3]. Snails of the genus *Bulinus* are also implicated in the transmission of bovine schistosomiasis; for example, *Schistosoma bovis* afflicts mainly cattle, causing productivity losses and thus economic losses. Hybridisation of *S. bovis* with *S. haematobium* results in offspring with an increased host range, altered virulence and hybrid vigour [6–8]. Therefore, a One Health approach is paramount for effective prevention and control of schistosomiasis.

**Fig. 1:**
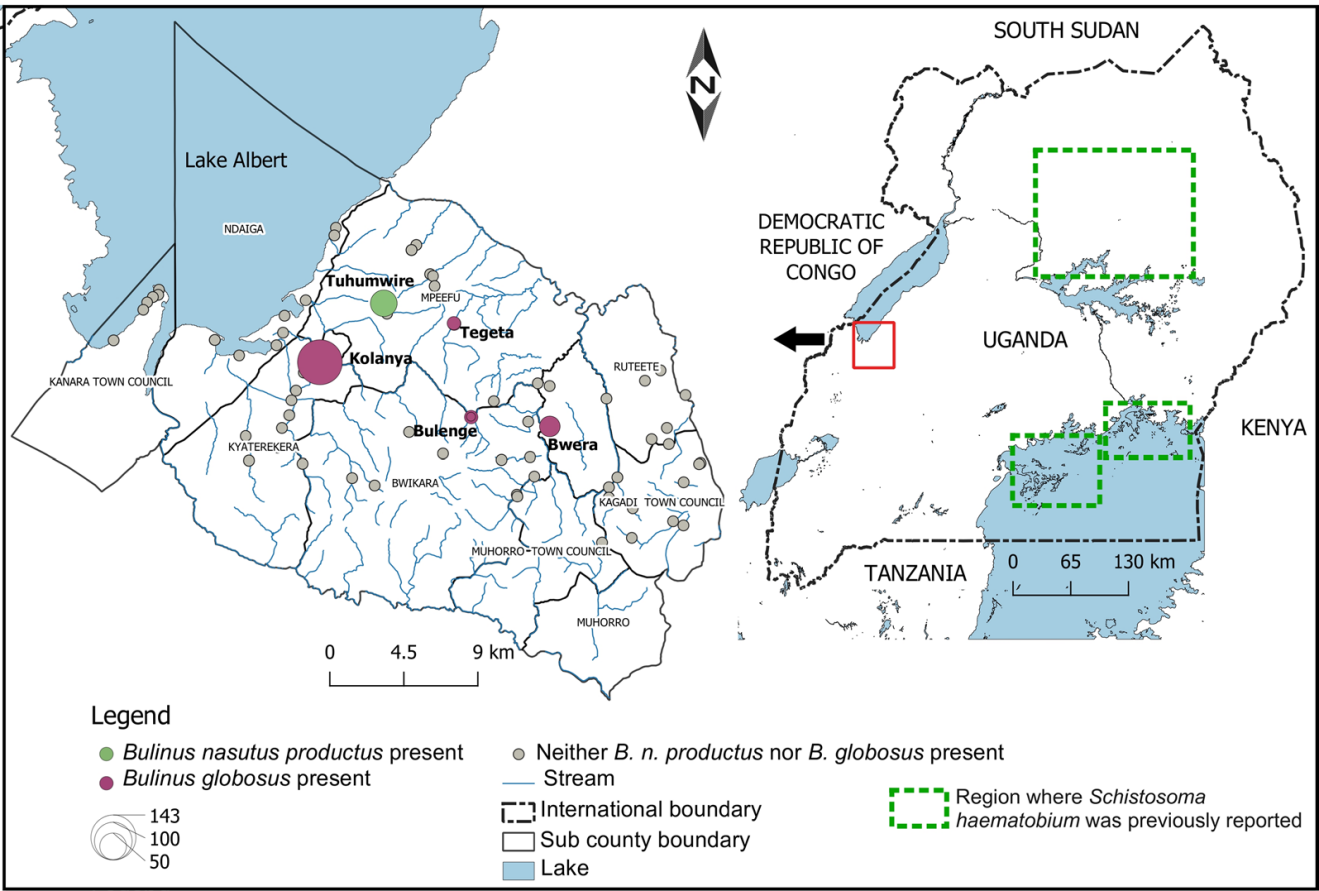
Distribution of the snails *Bulinus globosus* and *Bulinus nasutus productus* across 73 sites south-east of Lake Albert in Uganda sampled in October and November 2020. The maroon-coloured circles indicate locations where *B. globosus* was found, the green-filled circles represent the location of the Tuhumwire site where *B. nasutus productus* was found. The size of the circle represents the total number of snails collected. The locations where *Schistosoma haematobium* was previously reported [21] are indicated by green rectangles with dashed outlines. *Schistosoma haematobium* has also been reported in neighbouring countries, including the Democratic Republic of Congo, South Sudan, Kenya and Tanzania

In Uganda, intestinal schistosomiasis caused by *Schistosoma mansoni* is highly endemic and widespread, especially around the Great Lakes [9]. In contrast, urinary schistosomiasis is virtually absent, with the exception of a few cases in the central northern region of the country and, therefore, this infection is not considered to be of public health importance [10]. Nevertheless, the narrow geographical distribution of *S. haematobium* has been enigmatic and thought to be a result of the absence of suitable intermediate *Bulinus* snail hosts [11]. During a routine malacological survey, we encountered, unexpectedly, a high abundance of these intermediate host snails in the southern part of the Lake Albert region of Uganda (Fig. 1). The region is endemic to *S. mansoni*, with recent estimates indicating over 50% prevalence of intestinal schistosomiasis despite regular mass drug treatment [9]. Clearly, open defecation and urination behaviours are common practices in these communities, thereby also favoring the potential transmission of urinary schistosomiasis if the respective *Bulinus* species would be present. However, until now, there have been no reports of the latter nor of *S. haematobium* cases in this region.

## Methods

We conducted malacological surveys in 73 sites (Fig. 1) in October and November 2020. At each site, we recorded information on the locality, including human activities such as swimming, bathing, washing of household items, and animal grazing and watering, using an observation checklist in which the sex and number of people involved in the activity were noted at the time of snail collecting. The substrate was then actively searched for snails for 30 min by one person using a metallic sieve attached to a 2-m-long handle. The collected snails were placed in plastic containers, pooled per sampling site and transferred in ambient water to the laboratory where they were identified based on morphological characteristics [4]. Specimens were then individually placed in tissue culture plates to which mineral water was added. The plates were placed under artificial white light and checked after 2 h for emerging cercariae (parasitic larvae) under a stereo microscope. The snails were exposed to artificial light during daylight hours, usually between 10:00 am and 4:00 pm, to correspond to the natural patterns of parasite emergence [6]. Infected snails were individually placed in 2-ml tubes together with a subset of the emerged cercariae and fixed in 90% ethanol. The remaining snails were pooled per species/morphotype, per site, fixed in 90% ethanol and stored for DNA extraction at a later date. *Bulinus* snail morphotypes were selected and photographed before the entire snail tissue was separated from the shell and homogenised using a sterile scalpel; excess ethanol was removed using blotting paper. DNA was extracted using the E.Z.N.A. Mollusc DNA Kit protocol (OMEGA Bio-tek, Norcross, GA, USA), diluted with 1/10 ultrapure water and stored in the freezer at − 20 °C for later analysis.

The presence of *Schistosoma* spp. in the snail DNA extracts was determined using a two-step approach described by Schols et al. [12]. In the first step, termed infection rapid diagnostic PCR (RD-PCR), three markers were used to detect the presence of snail, trematode and *Schistosoma* spp. DNA concurrently. The performance of the PCR assays was compared with positive controls (DNA extract of adult worms) and a negative control (water). The second PCR, termed *Schistosoma* RD-PCR, discriminates among *Schistosoma* species and was only applied to samples that tested positive in the infection RD-PCR. A universal forward primer, Asmit1, was combined with species-specific reverse primers (Sh.R for *S*. *haematobium*, Sman.R for *S. mansoni*, Smat.R for *S. mattheei* and Sb.R for *S. bovis*/*S. curassoni*/*S. guineensis*), resulting in the amplification of cytochrome *c* oxidase subunit (*COX*1) gene fragments of different lengths depending on the parasite species. PCR products were run in a 3% agarose gel for visual interpretation.

Partial *COX*1 gene sequences for *Bulinus* snails were amplified using different primer combinations: the universal barcoding primers (LCO1490-HCO2198) [13], BulCox6-BulCox12 and Asmit1-Asmit2 [14]. Additionally, the entire internal transcribed spacer (*ITS*) region was amplified using the primers ETTS1 and ETTS2 [15]. The PCR reaction was performed according to the Qiagen™ Taq DNA polymerase Kit (Qiagen, Hilden, Germany) and the cycling conditions described by Kane et al. [14]. Specifically, 2.5 μl of DNA solution was added to 22.5 μl of the PCR master mix (2.5 μl PCR buffer ×10, 0.75 μl dNTP, 0.75 μl MgCl_2_, 1 μl of 10 µM primer1, 1 μl of 10 µM primer2, 0.15 μl TAQ, and 16.35 μl dH_2_O). The cycling conditions for *COX*1 and *ITS* region PCR reactions were: 94 °C for 5 min; 45 cycles of 94 °C for 15 s, 40 °C for 30 s and 72 °C for 45 s; and a final extension at 72 °C for 7 min. PCR fragments were separated in a 1% agarose gel, and the PCR products were purified for sequencing using the ExoSAP-IT™ PCR Product Cleanup Reagent (Thermo Fisher Scientific, Waltham, MA, USA) and then sequenced in both directions using the BigDye Terminator Protocol V3 (Qiagen). The forward and reverse DNA sequences were edited and aligned using Geneious Prime® version 2023.1.1 (Biomatters Ltd, Auckland, New Zealand). We then checked sequences for stop codons, indels and nuclear mitochondrial DNA sequences (NUMTs), which can be identified by reading frame shifts in the coding regions. To validate the species identities of the samples, we employed a BLAST (Basic Local Alignment Search Tool) search against the GenBank database. In addition, the following samples within the sub-Saharan region were retrieved from GenBank for phylogeny reconstruction: AM921839, AM921808, FN546814, OP233113, AM286294, ON117872, ON117894, AM921970 and MK414454 for *B. globosus*; AM286299 and AM921811 for *B. nasutus nasutus*; AM921815, OP233133, AM921989, AM921988 and AM286302 for *B. nasutus productus;* and AM286295 and AM286296 for *B. africanus* [5, 14, 16–18]. A multiple sequence alignment was constructed using the MUSCLE algorithm in MEGA X programme using default settings [19], which was trimmed to 631 bp for the ‘Folmer’ region, 360 bp for the ‘Asmit’ region and 439 bp for the *ITS*2 sequences. The best fitting nucleotide substitution model was chosen for the *COX*1 gene in MEGA X, based on the Bayesian information criterion (BIC) criterion. The phylogenetic relationships were inferred using maximum likelihood (ML) in MEGA X with 10,000 bootstrap replicates while uncorrected pairwise distance values (*p*-distances) were computed. *Biomphalaria sudanica* (OL423117 [20]) was used as an outgroup. A Chi-square (*χ*^2^) test was performed in R statistical software (v4.1.0; R Core Team 2021, R Foundation for Statistical Computing, Vienna, Austria) to compare snail abundance sampled in October and November.

## Results

A total of 267 individuals of *Bulinus globosus* (*n* = 188) and *B. nasutus productus* (*n* = 79) morphotypes were collected from five out of the 73 sampled sites (Fig. 1). The identity of *B. globosus* and *B. nasutus productus* collected at three and one sampling site(s), respectively, were confirmed by molecular barcoding (see below). At the Bulenge2 site, the *B. globosus* specimen was identified morphologically due to failure of DNA amplification.

*Bulinus globosus* was more abundant than *B. nasutus productus* and was collected from permanent stream points frequently used by humans and animals while *B. nasutus productus* was collected at a seasonal pond located in a communal livestock grazing field in Tuhumwire village (1.05143°N, 30.66487°E). More *B. globosus* snails were collected in November (*n* = 110) than in October (78; *χ*^2^ = 5.45, *p* = 0.02) while *B. nasutus productus* abundance remained constant in these two months (39 and 40, respectively; *χ*^2^ = 0.013, *p* = 0.91) (Table 1). None of the collected *B. globosus* snails shed cercariae, and RD-PCR results were negative for *Schistosoma* spp. infection. In contrast, two of the 32 (6.25%) *B. nasutus productus* snails shed *Schistosoma* sp. cercariae, with the prevalence increasing to 16% (4/25) when checked by infection RD-PCR. The infecting *Schistosoma* species was identified as *Schistosoma bovis* based on the *Schistosoma* RD-PCR.

**Table 1:** Observed human activities and abundance of snails at locations in the Lake Albert region of western Uganda where *Bulinus globosus* and *Bulinus nasutus productus* were sampled in October and November 2020

Site	GPS coordinates	Observed activities	*Bulinus globosus*		*Bulinus nasutus productus*		GenBank no. *COX*1		GenBank no. *ITS* rDNA
			October	November	October	November	‘Folmer’ region	‘Asmit’ region	
Bulenge	0.98913°N, 30.71254°E	Bathing, cattle drinking	0	2	0	0	_	_	_
Bwera	0.98388°N, 30.75554°E	Water collection, cattle drinking	20	10	0	0	OR553989; OR553992	OR553988	_
Kolanya	1.01909°N, 30.63013°E	Washing, water collection	54	89	0	0	OR553990; OR553991	OR553985; OR553986; OR553987	OR553999; OR554002
Tegeta	1.04047°N, 30.70325°E	Washing, water collection, swimming	4	9	0	0	_	OR553983; OR553984	_
Tuhumwire	1.05143°N, 30.66487°E	livestock grazing	0	0	40	39^a^	OR553993; OR553994; OR553995; OR553996	–	OR554000; OR554001; OR554003
		Total	78	110	40	39			

*COX1* Cytochrome *c* oxidase subunit gene,* ITS* internal transcribed spacer region,* rDNA* ribosomal DNA

The accession numbers of the sequenced samples for the partial *COX*1 and *ITS* rDNA regions are shown. For the ‘Folmer’ region, each species had only one haplotype while three haplotypes, one at each site, were obtained among *B. globosus* in the ‘Asmit’ region

^a^*B. nasutus productus* at Tuhumwire had a *Schistosoma bovis* prevalence of 6.25% (*n* = 32) and 16% (*n* = 25) as determined by shedding and diagnostic PCR techniques, respectively

**Fig. 2:**
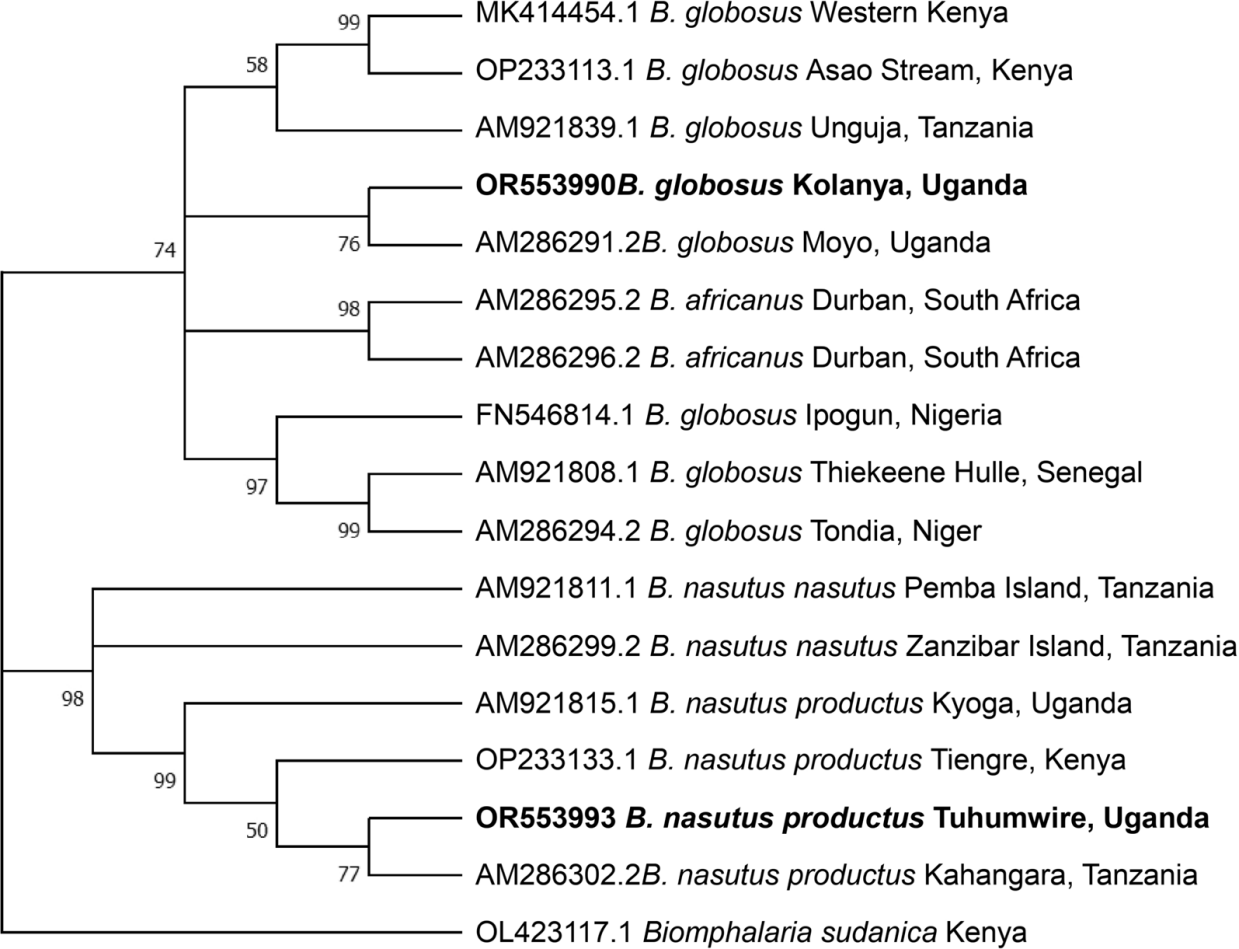
Phylogenetic relationships of *Bulinus globosus* and *Bulinus nasutus productus* from the Lake Albert region in this study (in bold) and GenBank reference sequences (with accession numbers, place and countries of origin where available) inferred using 631 bp of cytochrome *c* oxidase subunit 1 and maximum likelihood with the Hasegawa–Kishino–Yano model [22]. Only bootstrap values (10,000 replicates) > 50 are shown

A total of 11 *COX*1 sequences were generated for *B. globosus* across all sites. However, *B. globosus* DNA was challenging to amplify using the Folmer primers, with only four successful sequences, of which three were from snails from the same site (Kolanya). Primers targeting the Asmit region (Asmit1-Asmit2; 443 bp) were successfully used to amplify *B. globosus* from three of the four sites (Kolanya, *n* = 4; Tegeta, *n* = 2; Bwera, *n* = 1). Therefore, *B. globosus* at the Bulenge site was only identified morphologically. Different primer combinations were needed for certain isolates, as had been proposed by Kane et al. [14], which can probably be explained by variations in the primer binding site rather than PCR inhibitors since the internal control of the infection PCR (i.e.* 18S* snail marker) gave a positive signal and all samples had a DNA concentration > 7.6 ng/µl. Based on the partial *COX*1 sequences (631 bp from the Folmer region), one haplotype of *B. globosus* was identified that was highly similar (uncorrected *p*-distance = 0.16%) to the sequences from the Albert Nile (about 300 km away from our study area: AM9286291) in Moyo, Uganda, differing by three substitutions. This close relationship is also reflected in the phylogenetic tree (Fig. 2). Lower similarity was found with *B. globosus* haplotypes from Kenya (3.65% and 4.13% *p*-distance with reference sequences MK414454 and OP233113, respectively), Zanzibar Island (*p*-distance = 4.60%), Nigeria (*p*-distance = 4.92%) and Senegal (*p*-distance = 5.56%). However, higher variability was observed in the Asmit region. The intraspecific *p*-distances between the three obtained haplotypes ranged from 0.28% to 0.83% between the populations at Bwera and Tegeta, and Kolanya and Bwera, respectively. The haplotype at Bwera was identical to the haplotype from Kyaninga Crater Lake (ON112320). A similar phylogenetic clustering as in Fig. 2 was observed where the Ugandan population clustered with the East African populations (Additional file 1: Fig. S1), while *B. nasutus productus*, *B. africanus* and *B. globosus* from West Africa formed a second clade. However, the bootstrap support values were low, indicating low resolution in the Asmit alignment.

Seven identical *COX*1 sequences were obtained from *B. nasutus productus* from the Tuhumwire site using the Folmer *COX*1 primers. The intraspecific differences with GenBank sequences ranged from 0.31% with the Tanzanian population, 0.63% with the Cawente (Lake Kyoga-Ugandan) population, to 1.27% with the Kenyan populations. Generally, the *COX*1 phylogenetic analysis positioned both *B. globosus* and *B. nasutus productus* firmly within their respective species (Fig. 2). The *ITS*2 phylogenetic tree follows the same topology as the *COX*1 tree (Additional file 1: Fig. S2).

## Discussion

The aim of this study was to gain an understanding of the enigmatic absence of *S. haematobium* in the Lake Albert region of Uganda and specifically to investigate whether this absence results from the lack of suitable intermediate snail hosts. Our results indicate that the snails *B. globosus* and *B. nasutus productus* are present in the study area, with the former being both more widespread and more abundant in our study area than the latter. Similarly, the geographical distribution of *B. globosus* is known to be wider in sub-Saharan Africa compared to *B. nasutus productus* and even other hosts of *S. haematobium* [4], making it an important host of the disease. Notably, the *B. nasutus productus* specimens recorded from this study is the western-most case report in the known range of the species. Both species were present in relatively high numbers, suggesting that these are viable populations that have been sustained over time. The presence of three haplotypes of *B. globosus* collected at three locations, indicating the presence of distinct subpopulations with possibly different colonisation origins in the East African region, supports this notion of viable populations. Nevertheless, the isolates cluster closely with *B. globosus* populations from the East African region [14, 17, 18], confirming their close relationship and possible (recent) introduction from endemic regions of *S. haematobium*. However, our study recorded high genetic distance in the partial *COX*1 gene of *B. globosus* from the West African snail populations, similar to findings of other studies, when compared with the East African populations [5, 14, 18], indicating the complexity of the *B. globosus* group [23]. Moreover, Tumwebaze et al. [17] reported that the name “*Bulinus globosus*” was being used to group different species based on morphological similarity of the shell. For example, the GenBank sequence from Senegal with accession number AM921808 [14] used for comparison in the present study was considered to be a different species [17]. Our study, therefore, provides additional evidence for considering the re-evaluation of the taxonomy of the *B. globosus* species complex.

*Bulinus globosus* is by far the most important snail host of *S. haematobium* in Africa, occurring in most areas with active disease transmission [18, 24]. The absence of *S. haematobium* in *B. globosus* snails in the present study suggests the absence of infective larvae in the study area, despite our observations of the local populations coming into contact with water on a regular basis and showing open urination behaviours at snail-infested sites. Nevertheless, urinary schistosomiasis was reported in Lango region of Uganda north of Lake Kyoga (about 300 km away from our study area) as early as 1951, with a mean prevalence of 56.8% [25], and more recently in 2011, with a mean prevalence of 3.74% [10]. However, in the latter study [10], Adriko et al. did not find any *B. globosus* or *B. nasutus productus* specimens during their malacological surveys. They only identified, morphologically, *Bulinus tropicus* and *Bulinus forskalii*, which are not known to naturally transmit *S. haematobium* [4, 18]; none of these snails were shedding *S. haematobium* cercariae, despite urinary schistosomiasis cases detected in the community. Nevertheless, Kane et al. [14] previously identified *B. globosus* previously collected in the Moyo district, and confirmed this identification through molecular barcoding; the presence of this snail species could be responsible for *S. haematobium* transmission in the region. The rapidly changing situation of urogenital schistosomiasis is subject to discussion and partly attributable to mass drug administration (MDA). There is some evidence indicating a reduction in infection from 28.2% pre-MDA to 12.3% [26], potentially attributable to the reduction in the presence of the snail intermediate hosts.

Our current findings show that all conditions are ideal to support the transmission of urogenital schistosomiasis in Lake Albert hinterlands. The suitable snail hosts are present, as are risky public health practices, such as bathing and washing in rivers and ponds and open defecation/urination behaviours ([9]; Additional file 1: Figs. S1, S2), and the parasite is present in neighbouring regions and countries. It is a very dynamic area in terms of human mobility, with movement from neighbouring endemic regions and countries such as the Democratic Republic of Congo; consequently, introductions from these areas is possible. There are many cases of outbreaks of urinary and intestinal schistosomiasis following ecological changes and subsequent snail introduction, such as the construction of dams in Senegal [27] or the recent emergence of *S. mansoni* in Lake Malawi as a result of colonisation by *Biomphalaria pfeifferi* snails [28]. *Schistosoma mansoni* emergence quickly transitioned to an intestinal schistosomiasis outbreak in an area that was only known to be endemic for *S. haematobium* [29].

The partial *COX*1 sequences of *B. nasutus productus* obtained from this study is highly similar to the GenBank samples from Tanzania and Kenya. Natural transmission of *S. haematobium* by *B. nasutus productus* was recently reported in neighbouring Kenya [18] and previously in Tanzania [30], indicating that the Ugandan population could also support the transmission of urogenital schistosomiasis. However, to date, we have only found the presence of *S. bovis* cercariae in *B. nasutus productus* and no infections with *S. haematobium*, as confirmed by the RD-PCR tests*.* The prevalence of *S. bovis* was high (16% based on the infection PCR), indicating ongoing transmission of bovine schistosomiasis in cattle from the communal grazing fields where the snails were sampled. It is therefore important to investigate the extent of *S. bovis* distribution among livestock in the surrounding communities and the associated economic damage caused to the livestock industry in the area. This information should be shared with the respective communities together with possible treatment and prevention strategies.

## Conclusions

In summary, the presence of snail hosts in the Lake Albert region known to naturally transmit *S. haematobium* highlights the potential outbreak risk for urogenital schistosomiasis in the area. A better understanding of the dynamics of the snail population and status of parasite infection in space and time will provide further clarity into the exact magnitude of this risk. We therefore urge for increased snail monitoring and *S. haematobium* surveillance in the surrounding communities with the aim to rapidly detect a potential outbreak. Additionally, controlled snail infection experiments are needed to study the susceptibility of both snail species towards Ugandan *S. haematobium* isolates and those of neighbouring countries.

### Supplementary Information

**Additional file 1. Figure S1:** Phylogenetic relationships of *Bulinus globosus* from Lake Albert region (this study; in bold) and GenBank reference sequences (with accession numbers, place and countries of origin whenever available) inferred using 360 bp of the cytochrome *c* oxidase subunit I (Asmit region) and maximum likelihood with the Hasegawa–Kishino–Yano model + Gamma model [22]. Only bootstrap values (*n* = 10,000) above 50% are shown.** Figure S2:** Phylogenetic relationships of *Bulinus globosus* and *Bulinus nasutus productus* from Lake Albert region (this study; in bold) and GenBank reference sequences (with accession numbers, place and countries of origin whenever available) inferred using 439 bp of the nuclear internal transcribed spacer 2 (*ITS2*) and maximum likelihood with the Hasegawa–Kishino–Yano (HKY + I) model [22]. Only bootstrap values (*n* = 10,000) above 70% are shown.

## Data Availability

All of the data used in this paper are available in the article and Additional file 1: Figs. S1 and S2. All of the DNA sequences will be uploaded to the GenBank and accession numbers will be publicly available.

## References

[1] WHO. Schistosomiasis (Bilharzia. Schistosomiasis. 2023. https://www.who.int/health-topics/schistosomiasis#tab=tab_1. Accessed 26 Sept 2023.

[2] WHO. Control of neglected tropical diseases. 2023. https://www.who.int/teams/control-of-neglected-tropical-diseases/schistosomiasis/epidemiology. Accessed 27 Sept 2023.

[3] Aula OP, McManus DP, Jones MK, Gordon CA (2021). Schistosomiasis with a focus on Africa. Trop Med Infect Dis.

[4] Brown DS (1994). Freshwater snails of Africa and their medical importance.

[5] Zhang SM, Bu L, Lu L, Babbitt C, Adema CM, Loker ES (2022). Comparative mitogenomics of freshwater snails of the genus* Bulinus*, obligatory vectors of *Schistosoma haematobium*, causative agent of human urogenital schistosomiasis. Sci Rep.

[6] Savassi BAES, Mouahid G, Lasica C, Mahaman SDK, Garcia A, Courtin D (2020). Cattle as natural host for Schistosoma haematobium (Bilharz, 1852) Weinland, 1858 x Schistosoma bovis Sonsino, 1876 interactions, with new cercarial emergence and genetic patterns. Parasitol Res.

[7] Huyse T, Webster BL, Geldof S, Stothard JR, Diaw OT, Polman K (2009). Bidirectional introgressive hybridization between a cattle and human schistosome species. PLoS Pathog.

[8] Leger E, Webster JP (2017). Hybridizations within the Genus *Schistosoma*: implications for evolution, epidemiology and control. Parasitology.

[9] Exum NG, Kibira SPS, Ssenyonga R, Nobili J, Shannon AK, Ssempebwa JC (2019). The prevalence of schistosomiasis in Uganda: a nationally representative population estimate to inform control programs and water and sanitation interventions. PLoS Negl Trop Dis.

[10] Adriko M, Tinkitina B, Tukahebwa EM, Standley CJ, Stothard JR, Kabatereine NB (2018). Data on the pre-MDA and post MDA interventions for *Schistosoma mansoni* and *Schistosoma haematobium* in a co-endemic focus in Uganda: 1951–2011. Data Brief.

[11] Tumwebaze I, Clewing C, Dusabe MC, Tumusiime J, Rugunda GK, Hammoud C (2019). Molecular identification of *Bulinus* spp. intermediate host snails of *Schistosoma* spp. in crater lakes of western Uganda with implications for the transmission of the *Schistosoma haematobium* group parasites. Parasit Vectors.

[12] Schols R, Carolus H, Hammoud C, Mulero S, Mudavanhu A, Huyse T (2019). A rapid diagnostic multiplex PCR approach for xenomonitoring of human and animal schistosomiasis in a ‘One Health’ context. Trans R Soc Trop Med Hyg.

[13] Folmer O, Black M, Hoeh W, Lutz R, Vrijenhoek R (1994). DNA primers for amplification of mitochondrial cytochrome c oxidase subunit I from diverse metazoan invertebrates. Mol Mar Biol Biotechnol.

[14] Kane RA, Stothard JR, Emery AM, Rollinson D (2008). Molecular characterization of freshwater snails in the genus *Bulinus*: A role for barcodes?. Parasit Vectors.

[15] Kane RA, Rollinson D (1994). Repetitive sequences in the ribosomal DNA internal transcribed spacer of *Schistosoma haematobium*, *Schistosoma intercalatum* and *Schistosoma mattheei*. Mol Biochem Parasitol.

[16] Nalugwa A, Jørgensen A, Nyakaana S, Kristensen TK (2013). Evolutionary relationships of Bulinus (Gastropoda Planorbidae) shows the existence of 3 species complexes in the Albertine Rift freshwater bodies. Adv Anim Sci Theriogenol Genet Breed.

[17] Tumwebaze I, Clewing C, Chibwana FD, Kipyegon JK, Albrecht C (2022). Evolution and biogeography of freshwater snails of the genus *Bulinus* (Gastropoda) in afromontane extreme environments. Front Environ Sci.

[18] Babbitt CR, Laidemitt MR, Mutuku MW, Oraro PO, Brant SV, Mkoji GM (2023). *Bulinus* snails in the Lake Victoria Basin in Kenya: systematics and their role as hosts for schistosomes. PLoS Negl Trop Dis.

[19] Kumar S, Stecher G, Li M, Knyaz C, Tamura K (2018). MEGA X: molecular evolutionary genetics analysis across computing platforms. Mol Biol Evol.

[20] Laidemitt MR, Gleichsner AM, Ingram CD, Gay SD, Reinhart EM, Mutuku MW (2022). Host preference of field-derived *Schistosoma mansoni* is influenced by snail host compatibility and infection status. Ecosphere.

[21] London Applied & Spatial Epidemiology Research Group. Global Atlas of helminth infections. Distribution of schistosomiasis survey data in Uganda. https://www.thiswormyworld.org/maps/distribution-of-schistosomiasis-survey-data-in-uganda. Accessed 25 July 2023.

[22] Hasegawa M, Kishino H, Yano T (1985). Dating of the human-ape splitting by a molecular clock of mitochondrial DNA. J Mol Evol.

[23] Pennance T. Genetic diversity and evolution within the genus *Bulinus* and species-level interactions with the transmission of *Schistosoma haematobium* group parasites. Cardiff: Natural History Museum & Cardiff University; 2020.

[24] Pennance T, Ame SM, Amour AK, Suleiman KR, Muhsin MA, Kabole F (2022). Transmission and diversity of *Schistosoma haematobium* and *S. bovis* and their freshwater intermediate snail hosts *Bulinus globosus* and *B. nasutus* in the Zanzibar Archipelago, United Republic of Tanzania. PLoS Negl Trop Dis.

[25] Schwetz J (1951). On vesical Bilharzia in the lango district (Uganda). Trans R Soc Trop Med Hyg.

[26] Adriko M, Tinkitina B, Tukahebw EM, Standley CJ, Stothard JR, Kabatereine NB (2018). The epidemiology of Schistosomiasis in Lango Region Uganda 60 years after Schwetz 1951: Can Schistosomiasis be eliminated through mass drug administration without other supportive control measures?. Acta Trop.

[27] Talla I, Kongs A, Verlé P, Belot J, Sarr S, Coll AM (1920). Outbreak of intestinal schistosomiasis in the Senegal River Basin. Ann Soc Belg Med Trop.

[28] Alharbi MH, Condemine C, Christiansen R, Lacourse EJ, Makaula P, Stanton MC (2019). *Biomphalaria pfeifferi* Snails and Intestinal Schistosomiasis, Lake Malawi, Africa, 2017–2018. Emerg Infect Dis.

[29] Kayuni SA, O’Ferrall AM, Baxter H, Hesketh J, Mainga B, Lally D (2020). An outbreak of intestinal schistosomiasis, alongside increasing urogenital schistosomiasis prevalence, in primary school children on the shoreline of Lake Malawi, Mangochi District. Malawi Infect Dis Poverty.

[30] Mccullough FS, Eyakuze VM, Msinde J, Nditi H (1968). Water resources and bilharziasis transmission in the Misungwi area, Mwanza District, north-west Tanzania. East Afr Med J.

