# Supplementary material for: An accident waiting to happen? Exposing the potential of urogenital schistosomiasis transmission in the Lake Albert region, Uganda
Source: Parasit Vectors. 2023 Nov 3;16:398. doi: 10.1186/s13071-023-06017-3 (PMC10623741; doi:10.1186/s13071-023-06017-3)
Supplement: Supplementary file 1 — Additional file 1. Figure S1: Phylogenetic relationships of Bulinus globosus from Lake Albert region (this study; in bold) and GenBank reference sequences (with accession numbers, place and countries of origin whenever available) inferred using 360 bp of the cytochrome c oxidase subunit I (Asmit region) and maximum likelihood with the Hasegawa–Kishino–Yano model + Gamma model [22]. Only bootstrap values (n = 10,000) above 50% are shown. Figure S2: Phylogenetic relationships of Bulinus globosus and Bulinus nasutus productus from Lake Albert region (this study; in bold) and GenBank reference sequences (with accession numbers, place and countries of origin whenever available) inferred using 439 bp of the nuclear internal transcribed spacer 2 (ITS2) and maximum likelihood with the Hasegawa–Kishino–Yano (HKY + I) model [22]. Only bootstrap values (n = 10,000) above 70% are shown. [file 13071_2023_6017_MOESM1_ESM.docx]

**Supplementary Information**

**An accident waiting to happen? Exposing the potential for urogenital schistosomiasis transmission in the Lake Albert region, Uganda**

*Julius Tumusiime^1,2*^, Grace Kagoro-Rugunda^1^, Casim Umba Tolo^1^, Daisy Namirembe^1^, Ruben Schols^3,4^, Cyril Hammoud^3,5^, Christian Albrecht^2,1^ & Tine Huyse^3^*

^1^ Department of Biology, Mbarara University of Science and Technology, Uganda; ^2^ Institute of Animal Ecology and Systematics, Justus Liebig University Giessen, Germany; ^3^ Department of Biology, Royal Museum for Central Africa, Belgium; ^4^ Laboratory of Aquatic Biology, KU Leuven, Campus Kortrijk, Belgium; ^5^ Department of Biology, Ghent University, Belgium

* Email of the corresponding author: jtumusiime90@must.ac.ug


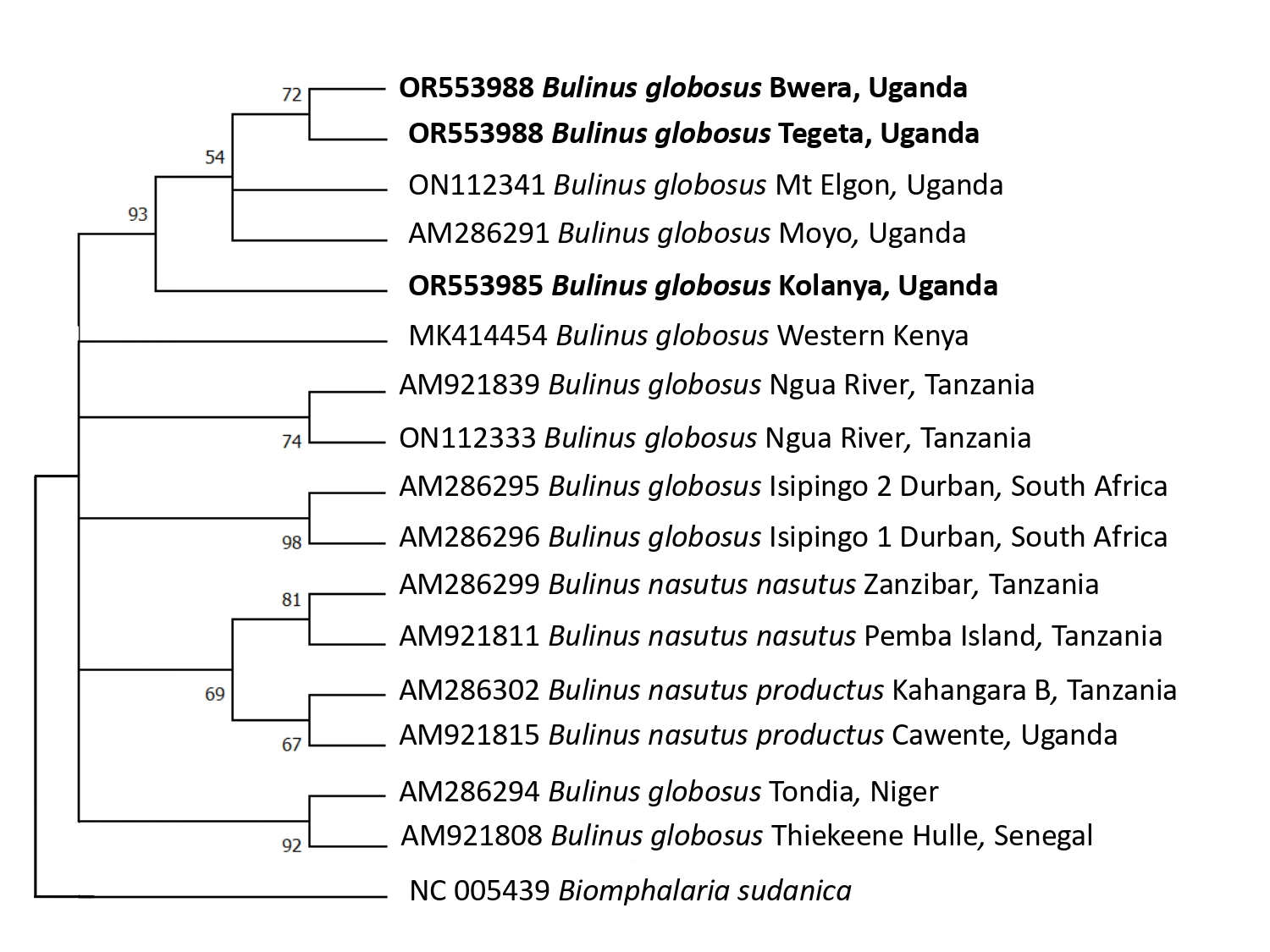


Supplementary Figure 1: Phylogenetic relationships of *Bulinus globosus* from Lake Albert region (this study; in bold) and GenBank reference sequences (with accession numbers, place and countries of origin whenever available) inferred using 360 bp of the cytochrome c oxidase subunit I (Asmit region) and Maximum Likelihood with the Hasegawa-Kishino-Yano model + Gamma model (Hasegawa et al., 1985). Only bootstrap values (n= 10,000) above 50% are shown.


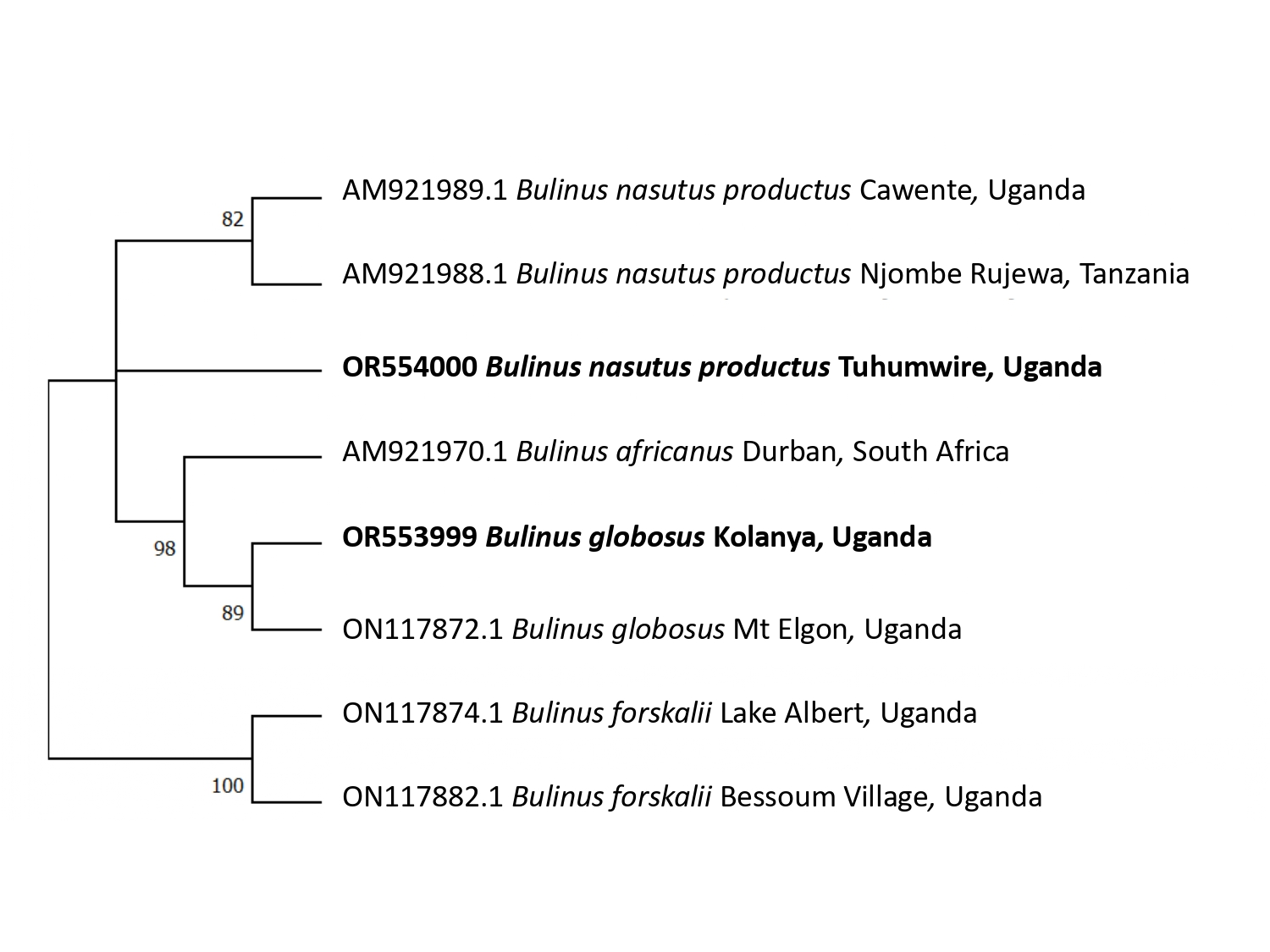


Supplementary Figure 2: Phylogenetic relationships of *Bulinus globosus* and *Bulinus nasutus productus* from Lake Albert region (this study; in bold) and GenBank reference sequences (with accession numbers, place and countries of origin whenever available) inferred using 439 bp of the nuclear internal transcribed spacer 2 (ITS2) and Maximum Likelihood with the Hasegawa-Kishino-Yano (HKY + I) model (Hasegawa et al., 1985). Only bootstrap values (n= 10,000) above 70% are shown.
